# Identification of the microRNA networks contributing to macrophage differentiation and function

**DOI:** 10.18632/oncotarget.8933

**Published:** 2016-04-22

**Authors:** Hong Zhou, Jie Zhang, Fiona Eyers, Yang Xiang, Cristan Herbert, Hock L. Tay, Paul S. Foster, Ming Yang

**Affiliations:** ^1^ Department of Respiratory Medicine, The Second Hospital, Jilin University, ChangChun, Jilin, People's Republic of China; ^2^ Priority Research Centre for Asthma and Respiratory Diseases, School of Biomedical Sciences and Pharmacy, Faculty of Health and Medicine, The University of Newcastle and Hunter Medical Research Institute, Callaghan, NSW, Australia; ^3^ Department of Physiology, Xiangya School of Medicine, Central South University, Changsha, Hunan, People's Republic of China; ^4^ Inflammation and Infection Research Centre, School of Medical Sciences, UNSW Australia, Sydney, Australia

**Keywords:** microRNA, macrophage, differentiation, transcriptional regulation, Immunology and Microbiology Section, Immune response, Immunity

## Abstract

Limited evidence is available about the specific miRNA networks that regulate differentiation of specific immune cells. In this study, we characterized miRNA expression and associated alterations in expression with putative mRNA targets that are critical during differentiation of macrophages. In an effort to map the dynamic changes in the bone marrow (BM), we profiled whole BM cultures during differentiation into macrophages. We identified 112 miRNAs with expression patterns that were differentially regulated 5-fold or more during BMDM development. With TargetScan and MeSH databases, we identified 1267 transcripts involved in 30 canonical pathways linked to macrophage biology as potentially regulated by these specific 112 miRNAs. Furthermore, by employing miRanda and Ingenuity Pathways Analysis (IPA) analysis systems, we identified 18 miRNAs that are temporally linked to the expression of CSF1R, CD36, MSR1 and SCARB1; 7 miRNAs linked to the regulation of the transcription factors RUNX1 and PU.1, and 14 miRNAs target the nuclear receptor PPARα and PPARγ. This novel information provides an important reference resource for further study of the functional links between miRNAs and their target mRNAs for the regulation of differentiation and function of macrophages.

## INTRODUCTION

Tissue macrophages are initially established during the embryonic period from progenitors derived from the yolk sac and foetal liver, these cells are largely replenished by circulating monocytes that originate from common myeloid progenitor cells in bone marrow [1–3]. Although many factors contribute to macrophage proliferation, macrophage colony-stimulating factor (M-CSF) is recognized as the most important factor that orchestrates not only differentiation, but also maturation [4–7]. During differentiation, M-CSF activates many intracellular transcriptional factors such as transcriptional factor PU.1 (PU.1), runt-related transcription factor 1 (RUNX1), CCAAT-enhancer-binding protein β, early growth response protein-1, interferon regulatory factor-1, nuclear transcription factor-Y and members of the Jun/Fos and Stat families [8]. Among these factors, PU.1 is essential and indispensable in macrophage proliferation by augmentation of CSF1R expression [9–12]. Furthermore, the expression of PU.1 is directly controlled by RUNX1, which belongs to a DNA-binding CBF-transcription factor family [13]. Interestingly, both PU.1 and RUNX1 are critical for haematopoiesis [13]. RUNX1 acts in concert with PU.1 to activate haematopoietic lineage development [14–16]. Likewise, the importance of RUNX1 in macrophage differentiation is well supported by the observation that deficiency in this transcription factor leads to severely impaired expression of macrophage related genes such as CSF1R, CSF2R and F4/80 [17]. Nevertheless, several lines of evidence suggest that a wide range of lineage-specific transcription factors and epigenetic factors such as microRNA (miRNA) may also participate in the mechanisms underlying macrophage development and activation [18–20]. As such, investigating the miRNAs networks involved in post-transcriptional regulation of gene expression, in the context of macrophage differentiation, will deepen our understanding of the mechanisms regulating this process and may lead to identifying new approaches for the treatment of macrophage-related diseases.

The biological importance of miRNAs is now widely recognized and many studies have demonstrated their importance in cellular differentiation and growth, as previously reviewed [21, 22]. In the immune system their importance for the regulation of immune cell differentiation and function is beginning to emerge [23–26], however the specific miRNA networks regulating translation during differentiation of specific subsets of leukocytes is yet to be fully characterised. In relation to monocyte/macrophage function, elevated expression of microRNA (miR) −21 has been shown to suppress the activation of NF-κB and the production of IL-6 in LPS-stimulated monocytic cells by binding to tumour-suppressor-programmed-cell-death protein 4 [27]. A recent study has shown that PU.1 activates a set of miRNAs that orchestrate macrophage differentiation, of which miR-146a directs the differentiation of tissue macrophages during adult/embryonic haematopoiesis [20]. Furthermore, miR-146a inhibits the response of human monocytic cells to LPS by downregulating Toll like receptor (TLR) signaling pathways [28]. These findings reflect the fact that miRNAs fine-tune cellular and tissue processes by binding to a range of transcripts, and their functional role is dependent on the transcriptional activity of a cell at a specific time. In this regard, miRNAs may act in concert with core transcriptional factors to activate macrophage differentiation and maturation, as observed in other cell types. However, few studies have attempted to investigate the global changes in miRNAs expression during macrophage differentiation and to correlate these changes with the levels of factors in pathways known to paly central roles in differentiation.

The aim of this study was to identify the predominant miRNAs involved in differentiation of macrophages by miRNA gene array and associate alterations in expression of these miRNAs with known factors that critically contribute to the out-growth of these cells from BM progenitors by employing various target prediction platforms. Our results identify the miRNA networks associated with macrophage differentiation and maturation, which provides an important platform for further functional investigations of these short regulatory RNAs in the generation and function of this leukocyte.

## RESULTS

### Generation of bone marrow derived macrophage (BMDM)

**Figure 1:**
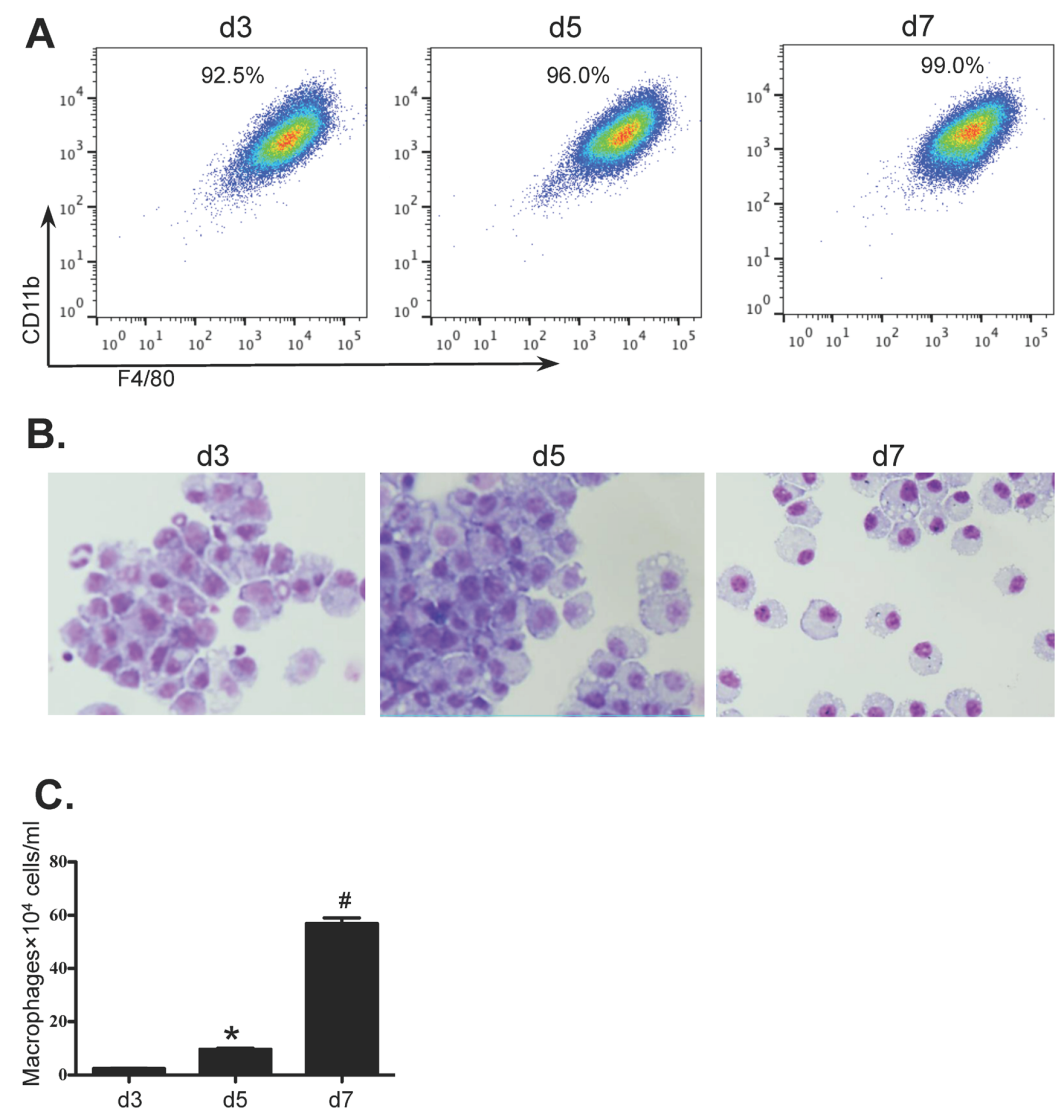
*In-vitro* differentiation of BMDM Bone marrow cells from BALB/c mice were cultured for 7 days (see Methods) and samples were collected on days 3, 5 and 7 from cultures grown in the presence of MCM. Macrophages were identified by **A.** flow cytometry (F4/80^+^CD11b^+^CD11c^−^Gr-1^−^), **B.** light microscopy with Giemsa staining (100×) and **C.** the numbers of BMDM were determined using a haemocytometer. Values are presented as mean ±SEM (*n* = 4~6), * *P* < 0.05 (v.s d3). # *P* < 0.05 (*vs* other groups).

Bone marrow cells from wild type BALB/c mice were isolated and cultured for 7 days to generate BMDM in the presence of macrophage conditioned medium (MCM). On days 3, 5 and 7, the percentages and morphological features of BMDMs were determined by flow cytometry and Giemsa staining. The numbers of BMDMs were also detrermined. The purity of BMDMs (F4/80^+^CD11b^+^CD11c^−^Gr-1^−^) was greater than 90% by day 3 of culture rising to 99% by day 7 (Figure 1A). Purity and morphology were also confirmed by Giemsa stained cytospins (Figure 1B). Although macrophages were almost non-detectable in the initial BM population, the numbers of cultured BMDMs were greatly increased from 2.44×10^4^±0.10×10^4^cells/ml on day 3 to 9.63×10 ^4^±0.42×10^4^cells/ml on day 5 and to 56.9×10^4^±2.16×10^4^cells/ml on day 7 (Figure 1C).

### MicroRNA profile of BMDM during differentiation

**Figure 2:**
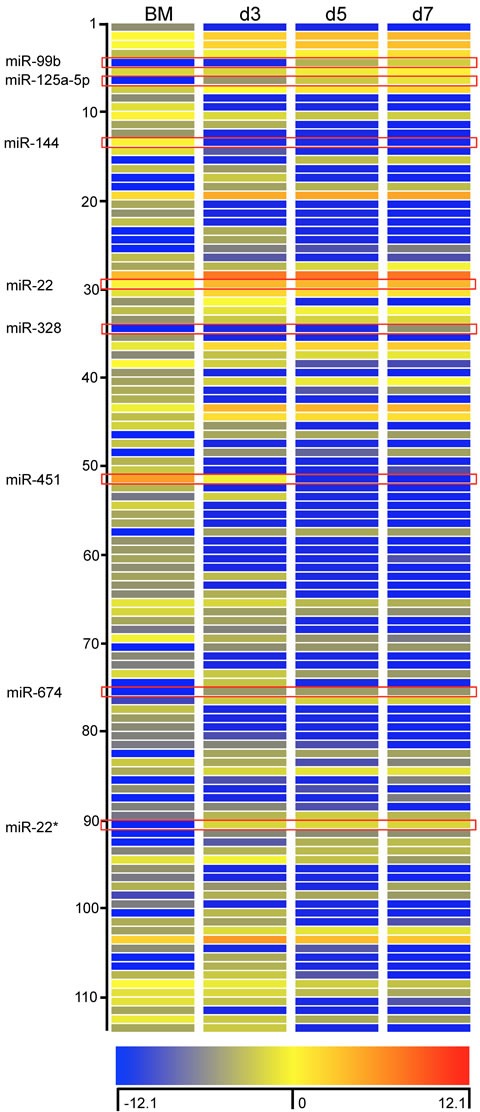
Characterization of miRNA expression during differentiation of BMDM Heat map representation of expression levels of miRNA that were up-regulated or down-regulated by more than 5-fold. The fluorescence index of each miRNA at different time-points was further normalized to that of the respective miRNAs in the control group (isolated bone marrow cells). The normalized microarray data were analyzed by GeneSpring (Agilent). Scale ranges from a signal value of −12.1(blue) to +12.1(red).

**Figure 3:**
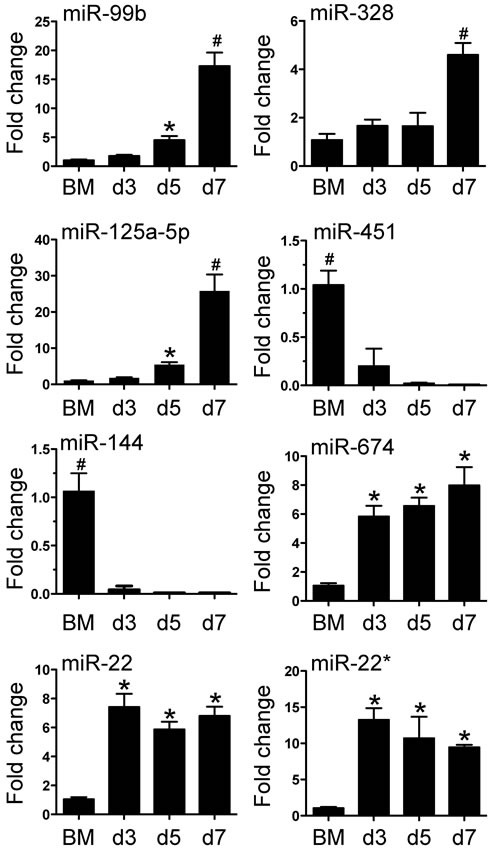
Verification of miRNA array expression data by Taqman quantitative PCR 8 miRNAs (miRNA −99b, −125a-5p, −144, −22, −328, −451, −674 and −22*) were selected to verify the changes in expression identified by the miRNA array. RNA was isolated from bone marrow cells or BMDM from day 3 to day 7. Values are presented as mean ±SEM (*n* = 4~6), * *P* < 0.05 (*vs*. BM), # *P* < 0.05 (*vs*. other groups).

As miRNAs are indispensible in post-transcriptional gene expression, we proceeded to characterise the miRNA profile of BM cells and in purified BMDM on days 3, 5 and 7. Total RNA was isolated from cells and hybridized to Agilent miRNA arrays as described in Methods. A total of 112 miRNAs were identified using GeneSpring software based on a cut-off point of 5-fold increase or decrease in expression during BMDM differentiation. Among these miRNAs, 56 miRNAs displayed decreased expression and 58 miRNA displayed increased expression on day 3; 66 miRNAs showed decreased expression and 48 miRNAs showed increased expression on day 5 and 7, respectively (Figure 2). Detailed information of these miRNAs is included in the Supplementary Table 1. We then confirmed the differential expression for 8 of 112 miRNA with qPCR (Figure 3). The 8 miRNAs were selected as expression was dramatically altered and were miRNAs that have been linked to leukocyte development and inflammation [29–33]. This demonstrated that alterations in the expression of miRNA detected by microarray could be substantiated by qPCR. Furthermore, we were able to measure and confirm the expression of miR-99b, miR-328 and miR-125a-5p in lung macrophages (Supplementary Figure 1).

### Molecules and canonical pathways associated with the differentiation and function of macrophage and potentially stimulated by the miRNAs

**Figure 4:**
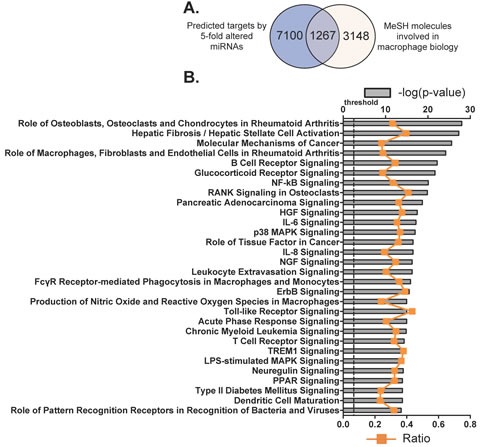
Potential molecules and canonical pathways predicted to be targeted by the miRNAs identified as differentially regulated during BMDM differentiation **A.** Target prediction by TargetScan database (http://www.targetscan.org/) was established on sequence data complementarity to target 3′UTR sites. Target molecules, associated with macrophage biology, were identified by exact syntax matching in the MeSH database. (http://www.nlm.nih.gov/MeSH/MeSHhome.html). **B.** Top 30 canonical pathways that consist of the putatively selected 1267 molecules as identified by IPA. The significance of the association between selected genes and the canonical pathway was evaluated by a right-tailed Fisher's exact test to calculate a *p*-value determining the probability that the association is not explained by chance alone (grey bars, upper y-axis). Ratios referring to the proportion of selected genes from a pathway related to the total number of molecules that make up that particular pathway were also displayed (line graph, bottom y-axis).

Firstly we identified the potential mRNA targets of the 112 differentially expressed miRNAs. TargetScan is a web-based application for predicting targets of miRNA in eukaryotes, by examining the conserved and/or non-conserved 7- and 8- mer sites that are located in the 3′-UTR regions of mRNAs and share sequence homology with corresponding miRNA(s). Predicted results are further graded by their probability of binding to the mRNA transcript [25, 26, 34]. We used the 95^th^percentile to predict the potential targets of these 112 miRNAs with TargetScan [35]. Consequently, the 3′-UTR regions of 8367 mRNA transcripts were identified as potential targets of this group of miRNAs (Figure 4A). By searching the MeSH database, these target mRNAs were further filtered in order to relate gene expression data with known macrophage-associated pathways, diseases and phenotypes. MeSH is one literature search tool for biomedical vocabularies, which links gene expression data with biological concepts such as pathways and disease phenotypes.

Eventually, 4415 mRNAs were identified by the MeSH database as comprising the exact terms “macrophages, GM-CSF, GM-CSF receptor, M-CSF receptor, scavenger receptor, myeloid progenitor cells and granulocyte-macrophage progenitor cells” with a link to at least one PubMed-affiliated reference. By plotting differences in distribution of mRNAs predicted by TargetScan and MeSH database, we revealed that 1267 of the original 8367 transcripts were known to be associated with macrophage biology and potentially bound by members of the 112 miRNAs by both search methodologies (Figure 4A and Supplementary Table 2).

**Figure 5:**
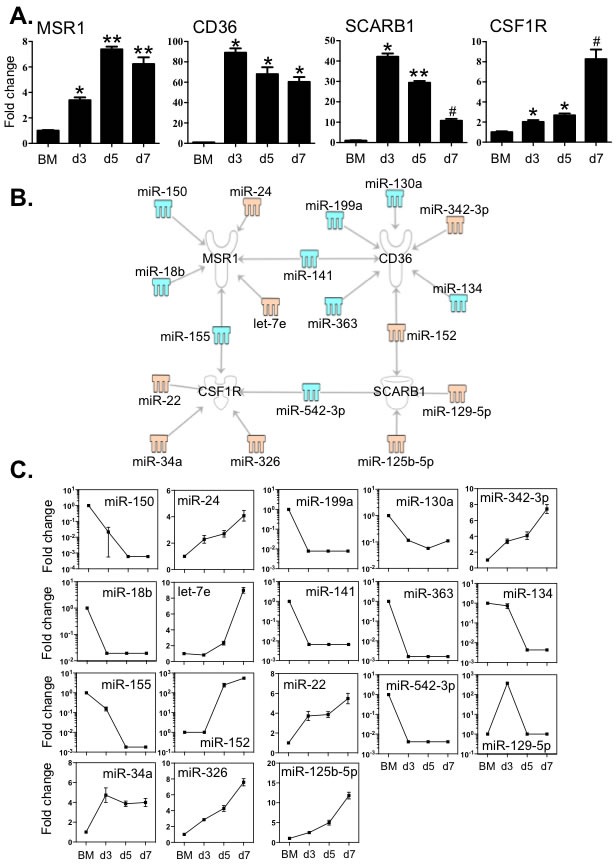
Expression levels of key macrophage receptors correlated with the expression of miRNAs that potentially target these transcripts **A.** Expression levels of MSR1, CD36, SCARB1 and CSF1R were determined by qPCR. **B.** Potential miRNAs targeting the 3′-UTR of MSR1, CD36, SCARB1 and CSF1R in BMDM cultures were identified by the TargetScan, and miRanda databases and IPA ingenuity system. Blue represents decreased expression of miRNAs, whereas yellow is for increased expressed miRNAs. **C.** The fold changes of potential regulating miRNAs were calculated based on the fluorescence index of each miRNA at different time-points, after normalization to that of the respective miRNAs in the control group (isolated bone marrow cells). Values are presented as mean ±SEM (*n* = 4~6), * *P* < 0.05 (*vs*. BM), ** *P* < 0.05 (*vs*. d3), # *P* < 0.05 (*vs*. other groups).

In an effort to further understand the way in which the 1267 macrophage-associated and miRNAs-target mRNAs correlate, these genes were then categorized according to signaling pathways by using IPA Ingenuity System and the top 30 canonical pathways were listed (Figure 5B; Supplementary Table 3). Among them, signaling pathways such as HGF, IL-6, NGF and ErbB are associated with cell death and survival; other pathways such as glucocorticoid receptor, NF-κB, RANK, p38 MAPK, IL-8, Nitric Oxide and reactive oxygen species, TLR, acute phase response and PPAR are linked to the development of inflammation. In total, there are 376 molecules (29.7% of the 1267 macrophage associated transcripts) that are involved in these top 30 canonical pathways. Many of the pathways underpin the mechanisms of cell proliferation and apoptosis, nevertheless these pathways may also participate in the regulation of macrophage differentiation and function. Collectively, these data indicate important roles of the identified miRNAs in the regulation of both the growth, function and cell survival of BMDMs.

### Multiple miRNAs are linked to the expression of key macrophage signature receptors

CSF1R predominantly activates the differentiation of macrophages and scavenger receptors such as MSR1, CD36 and Scavenger Receptor B1 (SCRAB1) are important for these innate immune cells to eliminate foreign substances and cellular debris. We then examined the expression of these molecules in BM cells and BMDM during culture by qPCR (Figure 5A). The transcripts of MSR1 increased greater than 4-fold, peaking at day 5. Expression levels of CD36, SCRAB1 and CSF1R were also markedly elevated compared to that of BM cells. The level of SCRAB1 gradually decreased after day 5, although the level at day 7 was still significantly higher than that in BM cells. These changes in expression corresponded to the significant increase in macrophage numbers between day 3 and day 7.

To examine whether any of the 112 differentially expressed miRNAs (Figure 2) might have binding sites in the target mRNA 3′-UTR region, we used the IPA system and the miRanda database to test the relationships between miRNAs and the specific transcripts (Figure 5B and 5C). MSR1 was linked to 6 miRNAs; expression of 4 of these miRNAs showed a decrease (miR −18b, −150, −141 and −155) and 2 (miR-24 and let-7e) were increased. CD36 may correlate to 7 miRNAs, of which (miR −130a, −134, −141, −199a and −363) were decreased and 2 (miR −152 and −342-3p) were increased (Figure 5B and 5C). Two miRNAs with increased expression (miR −125b-5p and −152) and another two miRNAs with decreased expression (miR −129-5p and −542-3p) are associated with the expression of SCRAB1 (Figure 5B and 5C). Five miRNAs (miR −22, −34a, −155, −326 and −542-3p) may correlate to macrophage differentiation as they are linked to CSF1R (Figure 5B and 5C). Among these miRNAs, four of them (miR −141, −152, −155 and −542-3p) are associated with multiple targets. The 3′-UTR binding sites of miRNAs are shown in Supplementary Table 4.

### Critical macrophage transcription regulators correlated to distinct sets of miRNAs

**Figure 6:**
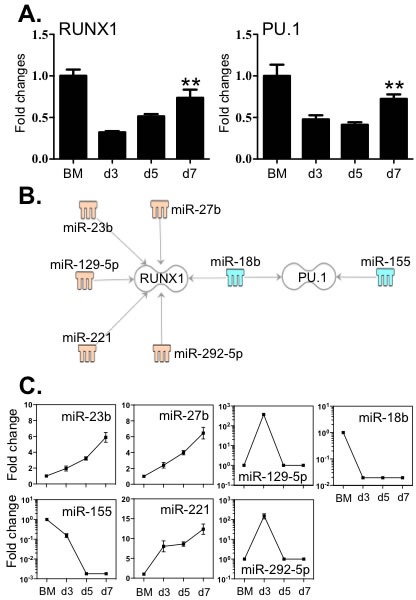
Expression of RUNX1 and PU.1 correlated with the expression levels of miRNAs that potentially target these transcripts **A.** Expression levels of RUNX1 and PU.1 were determined by qPCR. **B.** Potential miRNAs targeting the 3′-UTR of RUNX1 and PU.1 were identified by TargetScan, the Miranda database and IPA ingenuity system. Blue represents decreased expression of miRNAs, whereas yellow is for increased expressed miRNAs. **C.** The fold changes of potential regulating miRNAs were calculated based on the fluorescence index of each miRNA at different time-points, after normalization to that of the respective miRNAs in the control group (isolated bone marrow cells). Data represent three independent BMDM cultures. Values are presented as mean ±SEM (*n* = 4~6), ** *P* < 0.05 (*vs*. d3).

The lineage development of macrophages is critically determined by the coordinated action of RUNX1 and PU.1 [9, 13, 17]. Therefore, we evaluated the expression levels of these factors by qPCR and correlated them to the levels of the 112 miRNAs with greater than 5-fold alteration. Although the expression of RUNX1 and PU.1 in BM cells was significantly higher than in differentiating macrophages (reflecting their global role in haematopoiesis) (Figure 6A), the levels of both transcriptional factors were significantly increased in macrophages between day 3 and day 7. Five miRNAs (miR −23b, −27b, −129-5p, −221 and −292-5p) increased during BMDM culture, which are associated with RUNX1 (Figure 6B and 6C). By contrast, miR-18b and miR-155, which decreased greater than 100 fold, are linked to the regulation of PU.1 transcripts. Moreover, miR-18b is also associated with the regulation RUNX1 transcripts. The 3′-UTR binding sites of miRNAs are shown in Supplementary Table 5.

**Figure 7:**
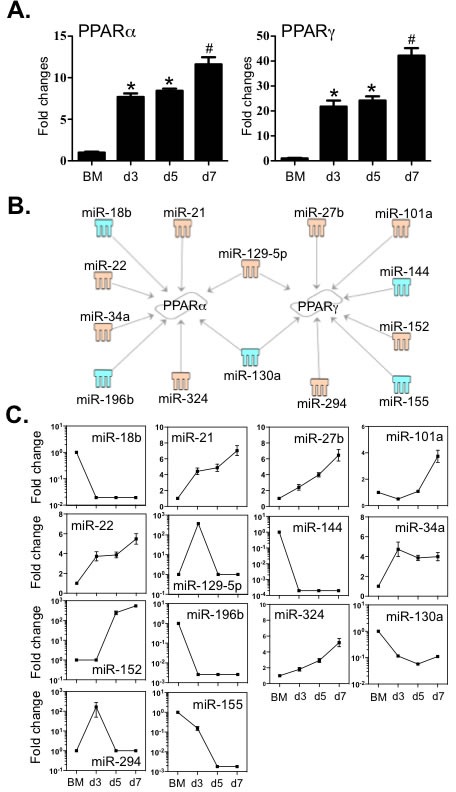
Expression of PPARα and PPARγ correlated to the expression levels of miRNAs that potentially target these transcripts **A.** Expression levels of PPARα and PPARγ were determined by qPCR. **B.** Potential miRNAs targeting the 3′-UTR of PPARα and PPARγ were identified by TargetScan and Miranda database and IPA ingenuity system. Blue represents decreased expression of miRNAs, whereas yellow is for increased expressed miRNAs. **C.** The fold changes of potential regulating miRNAs were calculated based on the fluorescence index of each miRNA at different time-points, after normalization to that of the respective miRNAs in the control group (isolated bone marrow cells). Values are presented as mean ±SEM (*n* = 4~6), * *P* < 0.05 (*vs*. BM), # *P* < 0.05 (*vs*. other groups).

PPARα and PPARγ are also involved in orchestrating the expression of genes that increase macrophage differentiation and function [36, 37]. By cross-comparison between TargetScan and the MeSH database, we examined the links between these two molecules and the 112 miRNAs. Interestingly, relative expression of transcripts encoding PPARα and PPARγ underwent significant increase during BMDM differentiation (Figure 7A). Two miRNAs, miR-129-5p and miR-130a, are differentially associated with both receptors (Figure 7B and 7C). In addition, six miRNAs are only linked to PPARα, among which four increased (miR −21, −22, −34a and −324) and two decreased (miR −18b and −196b). Another group of 6 miRNAs only correlated with PPARγ expression, among which four increased (miR −27b, −101a, −152 and −294) and two decreased (miR −144 and −155). The 3′-UTR binding sites of miRNAs are shown in Supplementary Table 6. Together, this data suggests that miRNAs establish a regulatory network contributing to the differentiation of macrophages by modulating these key transcriptional factors.

## DISCUSSION

In the present study, we have characterised the expression of miRNAs during the differentiation and maturation of BMDM. We observed a significant increase or decrease in the expression of 112 miRNAs. By employing TargetScan and the MeSH database, we identified 1267 mRNA transcripts that were potentially correlated to one or more of the 112 miRNAs and thus may contribute to macrophage maturation. Interestingly, fourteen of these miRNAs have also been observed in a previous study that examines the miRNA profiles in LPS-activated peritoneal derived macrophages [38]. Next, we characterised the expression of factors that are known to be intimately linked to the regulation of macrophages differentiation and function, such as the CSF1- and scavenger- receptors and transcriptional factors (e.g. PU.1 and RUNX1), and further correlated alterations in expression between these molecules and the 112 miRNAs. Through this analysis we identified a distinctive group of miRNA that are synchronously associated with macrophage differentiation and function.

Indeed, numerous myeloid cell derived cytokines may interact to define the final differentiated state of macrophages. For example, TNF-α, IL-1β and IL-6 are also closely involved in macrophage-regulated chronic inflammation [39, 40]. Both IL-12 and IL-23 may act in either an autocrine or paracrine manner to greatly enhance macrophage activation to control infections [41]. Even though at the early stage of differentiation, IL-1β, IL-3 and GM-CSF induce the proliferation of pluripotent myeloid precursors [8], it is M-CSF that plays a key role in determination of their differentiation to monocytic precursors, promonocytes and eventually macrophages [42]. Furthermore, PU.1 synchronizes with RUNX1 to contribute to differentiation by regulating CSF1R expression [43, 44]. In this regard, decreased miR-155 is associated with the levels of both CSF1R and PU.1, and miR-18b is also linked to both CSF1R and PU.1 (Figures 5 and 6), suggesting important roles for these two miRNAs in macrophage lineage development. Of note, miR-155 and miR-18b are further linked to regulation of PPARα and PPARγ transcript levels (Figures 6 and 7). Indeed, the activation of PPARγ has long been recognized to contribute to macrophage differentiation [45]. Unlike PPARγ, PPARα is not involved in macrophage differentiation, however both nuclear receptors are also important in maintaining the anti-inflammatory status of macrophages by negatively regulating the production of pro-inflammatory factors (e.g. IL-1β, IL-6 and TNFα) [37, 46, 47]. This suggests that downregulation of both miR-155 and miR-18b is important for regulating macrophage homeostasis. By contrast to the decreased expression of miR-155 and miR-18b, both miR-129-5p and miR-27b were only slightly increased during macrophage differentiation, although they were associated with alterations in the expression levels of RUNX1, PPARα and PPARγ. Indeed, an association between miR-27b and PPARγ was recently identified where this miRNA was shown to destabilize PPARγ transcripts [48]. Collectively our data suggests that multiple miRNAs function concomitantly to contribute to the expression of a range of target transcripts that correlate to macrophage differentiation.

In addition, our data suggest that many of the 112 differentially expressed miRNAs are involved in the regulation of multiple signaling pathways associated with the differentiation and function of macrophages (Figure 4). Among these miRNA-associated pathways, the glucocorticoid receptor and PPAR signalings belong to a nuclear receptor superfamily, which are important in the regulation of the proinflammatory and homeostatic status of macrophages [49]. Seed sequences for miRNA binding where also identified in signaling pathways and regulatory molecules such as Molecular Mechanisms of Cancer, HGF, NGF and ErbB, which promote the growth and survival of macrophages. Furthermore, the well-characterised pro-inflammatory molecules such as NF-κB, RANK, IL-6, p38 MAPK and IL-8, were also candidate targets. Molecules in pathways that are employed by macrophages to control infection such as FcγR-stimulated Phagocytosis in Macrophage/Monocyte, TLR, NO/ROS, LPS-stimulated MAPK, Acute Phase Response and Triggering Receptor Expressed on Myeloid Cells 1 (TREM1) were also potential targets of a number of the 112 miRNAs. As the afore-stated pathways are important components of macrophage associated immune responses, these data suggest important roles for miRNAs in the regulation of differentiation, cell death/survival and fundamental functions of macrophages.

We observed that the transcripts of three macrophage scavenger receptors including MSR1, CD36 and SCARB1 were potential targets of a set of 15 miRNAs (Figure 5). Notably a number of miRNA had multiple targets in the differentiation/maturation pathways: miR-155 and miR-18b were linked to the regulation of not only MSR1 but also PU.1 levels; miR-542-3p potentially linked to both SCRAB1 and CSF1R transcripts and CD36 levels are potentially associated with both miR-130a and miR-152 of which the former miRNA is linked to both PPARα and PPARγ regulation and the latter to PPARγ levels (Figures 5 and 7). These observations further suggest that miRNAs act in concert with their target mRNAs to maintain the required level of expression of macrophage-associated transcriptional factors and cell identity molecules to guide macrophage lineage development and maturation.

Regulation of mRNA transcription and translation depends on the temporal and spatial expression of an array of factors of which miRNAs are recognised as a key component. In this regard we have identified miRNAs that may be essential in macrophage differentiation and maturation by binding to key transcription factors and regulatory molecules. By using a range of data sources, including target information, expression profiles and literature validation, we have identified a network of 112 miRNAs that have the potential to correlate to key macrophage-related gene regulatory pathways. Our analysis provides insight into how specific miRNA may correlate to macrophage differentiation and function. By identification of these key miRNA networks and their potential targets we provide an important resource for more detailed investigation into the regulatory role of these miRNA networks in macrophage function.

## MATERIALS AND METHODS

### Animals

Wild type pathogen free BALB/c mice (6-8 weeks old) were obtained from the animal services unit of the University of Newcastle. All experiments were performed with approval from the Animal Ethics Committee of the University of Newcastle (A-2010-136).

### Culture and identification of BMDM

BMDM were differentiated and examined as previously described, with slight modifications [50, 51]. Briefly, mouse femurs were flushed with 3 ml ice cold HBSS through a 70 μm cell strainer. After lysis of red blood cells and washing with PBS, BM cells were cultured in petridishes for 7 days in DMEM/F12 medium (Life Technologies) supplemented with 10% FCS, 10 mM L-glutamine and 20% MCM (culture supernatant from L929 cell (ATCC, CCL-1), containing approximately 150 pg/ml M-CSF and no detectable GM-CSF) at a concentration of 4×10^4^cells/ml. Cells were then cultured at 37°C in a humidified atmosphere of 5% CO_2_ and 95% air. After 3, 5 and 7 days of culture, non-adherent cells were removed by multiple washes with PBS, and adherent samples were collected by centrifugation at 500× g for 10 min. The purity of BMDM was determined using Giemsa staining and flow cytometry.

### Giemsa staining

BMDM (2×10^5^cells) were centrifuged in 100 μl aliquots onto clean glass slides for 5 min at 300× g using a Cytospin centrifuge. Slides were air dried and fixed by submersion in methanol for 15 seconds followed by using Giemsa staining. Macrophages were determined morphologically as previously described [51, 52].

### Flow cytometry

Cells (3×10^5^cells) were incubated first with mouse Fc Receptor Block (2.4G2; BioXcell, West Lebanon, NH, USA) to inhibit non-specific binding of antibodies. After washing, cells were stained with anti-F4/80, anti-CD11b, anti-CD11c and anti-Gr-1' antibodies (BD Pharmingen). Numbers of positive cells were quantified by flow cytometry (FACSCanto flow cytometer, BD Biosciences, San Jose, CA). BMDMs were categorized by F4/80^+^CD11b^+^CD11c^−^Gr-1 cells [51, 52]. Data was collected on a FACS Canto flow cytometer and analysed with FlowJo software (TreeStar, Ashland, OR, USA).

### miRNA microarray

Total RNA was extracted from BM and BMDM cultures at days 3, 5 and 7 using Tri Reagent (Life Technologies) and miRNA microarray was carried out as previously reported [51, 52]. Briefly, the Agilent Spike-In control was added to 100 ng RNA, which was dephosphorylated by incubating the samples at 37°C for 30 minutes followed by ligation of Cy3 using the Complete Labelling and Hybridisation kit (Agilent). Following ligation and drying, the Cy3-labelled RNA samples were hybridized for 20h at 55°C to Agilent 8 × 15K mouse microRNA array slides AMADID 21828, which included 627 mouse miRNA and 39 mouse viral miRNA from the Sanger database 12.0. After washing with Agilent gene expression wash buffers, the hybridized microarrays were scanned on a High Resolution C scanner (Agilent). Data was extracted from scanned microarrays using Feature Extraction software version 10.7.3.1. The miRNAs at different time points were further normalized to the respective miRNAs in the control group (bone marrow cells only). The normalized microarray data was analyzed using GeneSpring software (Agilent).

### miRNA quantitative PCR

MiRNA qPCR reactions were performed using the TaqMan MicroRNA reverse transcription kit (Life Technologies), Taqman MicroRNA qPCR assays (Life Technologies) and TaqMan Universal PCR Master Mix, as previously described [25, 26]. Briefly, cDNA was reverse transcribed from total RNA using specific miRNA primers according to the suggested reaction conditions on a BioRad T100 thermal cycler. Quantitative polymerase chain reaction (qPCR) was performed using a ViiA 7 Real Time PCR System (Applied Biosystems, Carlsbad, CA). Sno202 was used as a housekeeping control RNA. Relative expression was calculated using the 2^−ΔΔCt^method.

### miRNA target analysis

For prediction of target mRNA of the differentially expressed miRNAs, we first used TargetScan 6.1 (http://www.targetscan.org/). MeSH database (http://www.nlm.nih.gov/mesh/meshhome.html) was employed to identify the molecules relevant to macrophage biology by exact matching [25, 26]. Then, miRanda (http://www.microrna.org/) analysis was employed to refine the predicted targets. Ingenuity Pathways Analysis (Ingenuity Systems, Redwood City, CA) software was used to identify canonical signaling pathways containing the miRNA-associated macrophage-relevant molecules and to establish the connection between miRNAs and their respective targets.

### mRNA Quantitative PCR

The method for quantitative PCR has been described in detail elsewhere [52]. Briefly, total RNA was isolated from BMDM culture at days 3, 5 and 7 with Tri Reagent (Life Technologies) and reverse transcribed to cDNA using M-MLV reverse transcriptase (Life Technologies). Q-PCR was performed using a ViiA 7 Real-Time PCR System. Amplicons were detected using SYBR green and expression was normalized to hypoxanthine-guanine phosphoribosyl transferase (HPRT). Primers sequences are shown in Supplementary Table 7.

### Statistical analysis

An initial one-way ANOVA followed by post hoc Bonferroni correction was used to test differences between means of groups. Values are reported as the means ± SEM for each experimental group. The number of samples at each time-point ranged from 4 to 6. Differences in means were considered significant if *p* was < 0.05.

## SUPPLEMENTARY MATERIALS FIGURES AND TABLES






