# Supplementary material for: Identification of the microRNA networks contributing to macrophage differentiation and function
Source: Oncotarget. 2016 Apr 22;7(20):28806–20. doi: 10.18632/oncotarget.8933 (PMC5045358; doi:10.18632/oncotarget.8933)
Supplement: Supplementary file 2 [file oncotarget-07-28806-s002.pdf]

# Identification of the microRNA networks contributing to macrophage differentiation and function

## Supplementary Material

**Supplementary Table 4: The binding potentials between MSR1, CD36, SCARB1 and CSF1R, and their respective miRNAs**

<table>
<tr><th>Target Gene</th><th>microRNAs</th><th>Binding Sites</th></tr>
<tr><td rowspan="8">MSR1</td><td>miR-141</td><td>3' gguAGAAAUGGUC-UGUC-ACAAu 5'<br>|:|||| ||| |||| ||||<br>1458:5' uuuUUUUUAACAGAACAGCUGUUc 3'</td></tr>
<tr><td rowspan="2">let-7e</td><td>3' uugaUAUGUUGGAGGAUGGAGu 5'<br>||:: | :|||||||||<br>1341:5' gaucAUGUCAAUUCCUACCUCa 3'</td></tr>
<tr><td>3' uuGAUAUGUUGGAGGAUGGAGu 5'<br>:|||| :||: :||||||<br>568:5' cuUUAUAAGACUAAUUACCUCu 3'</td></tr>
<tr><td>miR-155</td><td>3' ugGGGAUAGUGUUAAUCGUAAUu 5'<br>||||| :: |||||||<br>1251:5' aaCCCUAAAGUCUGCAGCAUUAa 3'</td></tr>
<tr><td>miR-150</td><td>3' gugaccauGUUCC--CAACCCUCu 5'<br>||| | |||||||<br>1887:5' gaauuagcCAAUGAUUUUGGGAGu 3'</td></tr>
<tr><td>miR-24</td><td>3' gacaaggacGACUU-GACUCGGu 5'<br>| ||| |||||||<br>1740:5' uaugcaggaCAGAACCUGAGCCc 3'</td></tr>
<tr><td>miR-18b</td><td>3' gauugucgugaucuaCGUGGAAu 5'<br>|||||||<br>260:5' gacucccauccccugGCACCUUu 3'</td></tr>
<tr style="display:none"><td></td><td></td></tr>
<tr><td rowspan="4">CD36</td><td>miR-130a</td><td>3' uacgGGAAAAUUGU-----AACGUGAc 5'<br>::||||||:| |||||||<br>239:5' aauaUUUUUUAAUAAAAUCUUGCACUu 3'</td></tr>
<tr><td rowspan="2">miR-342-3p</td><td>3' ugcccacgcuaaaGACACACUCu 5'<br>| |||||||<br>1138:5' aauuuuauagcagCAGUGUGAGa 3'</td></tr>
<tr><td>3' ugcccacgcuaaagacACACUCu 5'<br>||||||<br>518:5' aaaugaacaauucacaUGUGAGc 3'</td></tr>
<tr><td>miR-152</td><td>3' ggUUCAAGACAGUACG-UGACu 5'<br>|| || || | ||| ||||<br>452:5' gcAAAUU-UGGCUUGCAACUGu 3'</td></tr>
</table>

<table>
<tr><td rowspan="9"></td><td rowspan="2">miR-199a-5p</td><td>3' cuuguccaucagacuUGUGACCc 5'<br>|||||||<br>7:5' gaugagccuacauauACACUGGc 3'</td></tr>
<tr><td>3' cuuguccaucagacuuGUGACCc 5'<br>||||||<br>389:5' aauuuuuguuuucccuCACUGGa 3'</td></tr>
<tr><td rowspan="2">miR-363</td><td>3' auGUCUACCUAUG-GCACGUUAa 5'<br>:| ||  |||: : ||||||<br>175:5' ugUAAAUAUAUAUAUAUGCAAUa 3'</td></tr>
<tr><td>3' augucuaccuauggcACGUUAa 5'<br>||||||<br>1096:5' ucugcuaugucuuaaUGCAAUu 3'</td></tr>
<tr><td rowspan="2">miR-141</td><td>3' gguagaaauggucUGUCACAAu 5'<br>|:||||||<br>807:5' uuggagagcuguuAUAGUGUUa 3'</td></tr>
<tr><td>3' ggUAGAAAUGGUCUGUCACAAu 5'<br>:|:|||  :||  ||||||<br>1037:5' uuGUUUUUUGUAG-AAGUGUUc 3'</td></tr>
<tr><td>miR-134</td><td>3' ggggagaccaGUUGGUCAGUGu 5'<br>|||: ||||||<br>183:5' uauauauaugCAAUAAGUCACa 3'</td></tr>
<tr><td rowspan="6">SCARB1</td><td>miR-152</td><td>3' gguuCA-AGACAGUAC-GUGACu 5'<br>|| |||| :||| |||||<br>510:5' uaucGUCUCUGCUAUGUCACUGa 3'</td></tr>
<tr><td rowspan="2">miR-125b-5p</td><td>3' aguguucaaucccaGAGUCCCu 5'<br>|||||||<br>171:5' augugcaaaaacaaCUCAGGGa 3'</td></tr>
<tr><td>3' agUGUUCA--AUCC-CAGAGUCCCu 5'<br>| |:||   |||   |||||||<br>550:5' gcAGAGGUGGCAGGCCCCUCAGGGu 3'</td></tr>
<tr><td>miR-129-5p</td><td>3' cgUUCGGGUCUG---GCGUUUUUc 5'<br>| ||||| :|   :|||||||<br>159:5' ggAUGCCCACGCAUGUGCAAAAAc 3'</td></tr>
<tr><td rowspan="2">miR-542-3p</td><td>3' aaaGUCAAUAG-UUAGACAGUGu 5'<br>|||  : | :|||||||||<br>381:5' ccaCAGAGGACGGAUCUGUCACu 3'</td></tr>
<tr><td>3' aaagucaauaguuagACAGUGu 5'<br>||||||<br>509:5' cuaucgucucugcuaUGUCACu 3'</td></tr>
<tr><td rowspan="2">CSF1R</td><td>miR-155</td><td>3' ugGGGAUAGUGUUAAUCGUAAUu 5'<br>| :||  || |  |||||||<br>629:5' aaCAUUA-AACUAACAGCAUUAa 3'</td></tr>
<tr><td>miR-34a</td><td>3' uguuGGUC---GAUUC--U-GUGACGGu 5'</td></tr>
</table>

| | | |
|---|---|---|
| | | \|\|\|\|    \|\|::\|   \| \|\|\|\|\|\|\|<br>160:5' guucCCAGAGCCUGGGCCAUCACUGCCa 3' |
| | miR-22 | 3' ugUCAAGAAGUUGA----CCGUCGAa 5'<br>:\| \|\|\|\|  \|\|\|     \|\|\|\|\|\|\|<br>482:5' cuGGAUCUUGUACUGAGCGGCAGCUa 3' |
| | miR-326 | 3' ugaccuccuucccGGGUCUCc 5'<br>\|\|\|\|\|\|\|<br>150:5' ggccccguuuguuCCCAGAGc 3' |
| | miR-542-3p | 3' aaagucaauaguuaGACAGUGu 5'<br>\|\|\|\|\|\|\|<br>514:5' uucuacccagugcccUGUCACu 3' |

**Supplementary Table 5: The binding potentials between RUNX1 and PU.1, and their respective miRNAs**

<table>
<tr><th>Target Gene</th><th>microRNAs</th><th>Binding Sites</th></tr>
<tr><td rowspan="7">RUNX1</td><td>miR-292-5p</td><td>    3' guuUUCUCGGGGGUC-AAACUCa 5'<br>          ||| |: : :|| ||||||<br>327:5' uuuAAGUGUGUAUAGAUUUGAGc 3'</td></tr>
<tr><td rowspan="2">miR-27b</td><td>    3' cgucuugaaucggUGACACUu 5'<br>                    |||||||<br> 86:5' aucccggagggaaACUGUGAa 3'</td></tr>
<tr><td>    3' cgucUUGAAUCG--GUGACACUu 5'<br>           :|:|||||  ::||||||<br>110:5' uucuGAUUUAGCAAUGCUGUGAa 3'</td></tr>
<tr><td>miR-18b</td><td>    3' gauugucgugaucuaCGUGGAAu 5'<br>                      |||||||<br>261:5' ggaguuuucuuuuccGCACCUUa 3'</td></tr>
<tr><td>miR-23b</td><td>    3' ccaUUAGGG---ACCGUU-ACACUa 5'<br>          ||||||    || || |||||<br> 82:5' aagAAUCCCGGAGGGAAACUGUGAa 3'</td></tr>
<tr><td>miR-129-5p</td><td>    3' cguUCGGGU--CUGGCGUUUUUc 5'<br>          | |::|  ||::|||||||<br>275:5' cgcACCUUAUCGAUUGCAAAAAu 3'</td></tr>
<tr><td>miR-221</td><td>    3' cuUUGGGUCGUCUG---UUACAUCGa 5'<br>         |||::  :||||    |||||||<br>413:5' uuAACUU-UUAGACUUUCAUGUAGCu 3'</td></tr>
<tr><td rowspan="2">PU.1</td><td>miR-18b</td><td>    3' gauuGUCGUGAUCUACGUGGAAu 5'<br>           ::||:||:   |||||||<br>253:5' agucUGGCGCUG---GCACCUUu 3'</td></tr>
<tr><td>miR-155</td><td>    3' ugGGGAUAG-UGUUAAUCGUAAUu 5'<br>         ||| : | :| | ||||||||<br> 28:5' gaCCCCGCCGGCCA-UAGCAUUAa 3'</td></tr>
</table>

**Supplementary Table 6: The binding potentials between PPARα and PPARγ, and their respective miRNAs**

<table>
<tr><th>Target Gene</th><th>microRNAs</th><th>Binding Sites</th></tr>
<tr><td rowspan="12">PPARα</td><td rowspan="2">miR-130a</td><td>3' uacGGGAAAAUUG--UA-ACGUGAc 5'<br>||||   |||  || ||||||<br>391:5' aauCCCUGAAAACUAAUCUGCACUu 3'</td></tr>
<tr><td>3' uacGGGAAAAUUG-UA-ACGUGAc 5'<br>||||| |::| || ||||||<br>362:5' ccaCCCUUCUGGCUAUGUGCACUc 3'</td></tr>
<tr><td rowspan="3">miR-129-5p</td><td>3' cguucgggUCUGGCGUUUUUc 5'<br>| :: |||||||<br>461:5' ccauuuaaAUGUGGCAAAAAa 3'</td></tr>
<tr><td>3' cguucgggucuggcGUUUUUc 5'<br>||||||<br>918:5' caaaaacaaacaaaCAAAAAa 3'</td></tr>
<tr><td>3' cguucgggucuggcGUUUUUc 5'<br>||||||<br>898:5' ugcuuuguaaaaaaCAAAAAc 3'</td></tr>
<tr><td>miR-34a</td><td>3' ugUUGGUCGAUUCUGUGACGGu 5'<br>| |||| | :|||||||||<br>1383:5' ugAUCCAGAU-GGACACUGCCa 3'</td></tr>
<tr><td>miR-324</td><td>3' ugaaugacgaagguGAAAGGAu 5'<br>|||||||<br>1:5' -------------uCUUUCCUg 3'</td></tr>
<tr><td>miR-21</td><td>3' agUUGUAGUCAGACUAUUCGAu 5'<br>:| | :|||:|||||||||<br>557:5' ucGAGAAUAGUUUGAUAAGCUa 3'</td></tr>
<tr><td>miR-22</td><td>3' ugucaaGAAGUUGACCGUCGAa 5'<br>| ||  :||||||||<br>857:5' uaggccCCUCCUUUGGCAGCUc 3'</td></tr>
<tr><td rowspan="2">miR-18b</td><td>3' gaUUGUCGUGAUCUACG-UGGAAu 5'<br>:| |||| |:   || |||||<br>1194:5' ggGAAAGCAGUGCUGGCUACCUUc 3'</td></tr>
<tr><td>3' gauuGUCGUGAUCUACGUGGAAu 5'<br>||| :| :| ||||||||<br>1293:5' auuuCAGAGCAGGUUGCACCUUc 3'</td></tr>
<tr><td rowspan="2">miR-196b</td><td>3' ggguuguuguccUUUGAUGGAu 5'<br>|:|||||||<br>162:5' ugggcacuucuaAGACUACCUg 3'</td></tr>
<tr><td>3' gggUUGUUGUCCU--UUGAUGGAu 5'<br>:| |:||| :  ::||||||<br>1193:5' gggGAAAGCAGUGCUGGCUACCUu 3'</td></tr>
<tr><td>PPARγ</td><td>miR-27b</td><td>3' cgUCUUGAAUCGGUGACACUu 5'<br>|||| ||    :|||||||<br>78:5' uaAGAAAUU---UACUGUGAa 3'</td></tr>
</table>

| | | |
|---|---|---|
| | | |
| | miR-130a | 3' uacGGGAAAAUUG-UAACGUGAc 5'<br>:|||| || : ||||||||<br>27:5' uguUCCUUCUAUUGAUUGCACUa 3' |
| | miR-155 | 3' ugggGAUAGUGUUAAUCGUAAuu 5'<br>||:| | || ||||||<br>85:5' uuuaCUGUGAAAA--AGCAUUua 3' |
| | miR-129-5p | 3' cguucgggucuggcGUUUUUc 5'<br>||||||<br>96:5' aaagcauuuaaaaaCAAAAAg 3' |
| | miR-144 | 3' ucauguaguagauAUGACAu 5'<br>||||||<br>74:5' caccuaagaaauuUACUGUg 3' |
| | miR-101a | 3' aagucaauagugucAUGACAu 5'<br>||||||<br>73:5' acaccuaagaaauuUACUGUg 3' |
| | miR-152 | 3' gguucAAGACAGU--ACGUGAcu 5'<br>|||| |:: ||||||<br>28:5' guuccUUCUAUUGAUUGCACUau 3' |
| | miR-294 | 3' uguGUGUUUUCCCUUCGUGAAa 5'<br>::::||| |||||:||<br>87:5' uacUGUGAAA---AAGCAUUUa 3' |

## Supplementary Table 7: Primer pairs

| Primer Name | Direction | Sequence (5’-3’) |
|---|---|---|
| HPRT | Forward | AGGCCAGACTTTGTTGGATTTGAA |
| | Reverse | CAACTTGCGCTCATCTTAGGCTTT |
| CD36 | Forward | CAAGCTCCTTGGCATGGTAGA |
| | Reverse | TGGATTTGCAAGCACAATATGAA |
| MSR1 | Forward | TTAAAGGTGATCGGGGACAAA |
| | Reverse | CAACCAGTCGAACTGTCTTAAG |
| SCRAB1 | Forward | TTAAAGGTGATCGGGGACAAA |
| | Reverse | AGCTGATCTTAAAAGGGTCTTG |
| CSF1R | Forward | CGAGGGAGACTCCAGCTACA |
| | Reverse | GACTGGAGAAGCCACTGTCC |
| RUNX1 | Forward | ACTTCCTCTGCTCCGTGCTA |
| | Reverse | CGCGGTAGCATTTCTCAGTT |
| PPARα | Forward | GGAAAGACAACGGACAAATC |
| | Reverse | AAACTGGCACCCTTGAAAAAT |
| PPARγ | Forward | TGTGAGACCAACAGCCTGAC |
| | Reverse | TGATCGCACTTTGGTATTCTTGG |
| PU.1 | Forward | TGTCCACAACAACGAGTTTGAGAA |
| | Reverse | GGGACAAGGTTTGATAAGGGAAGC |
