# Supplementary material for: Identification of the microRNA networks contributing to macrophage differentiation and function
Source: Oncotarget. 2016 Apr 22;7(20):28806–20. doi: 10.18632/oncotarget.8933 (PMC5045358; doi:10.18632/oncotarget.8933)
Supplement: Supplementary file 3 [file oncotarget-07-28806-s003.pdf]

Identification of the microRNA networks contributing to macrophage differentiation and function

Supplementary Material

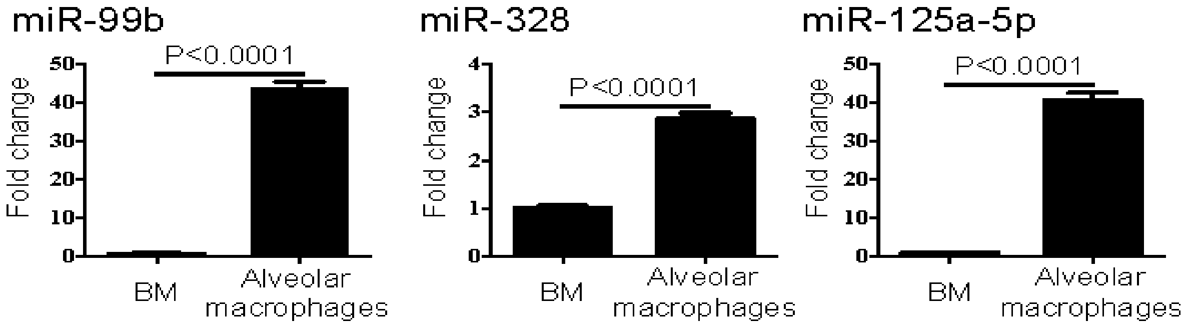

Supplementary Figure 1. Expression of miR-99b, miR-328 and miR-125a-5p in mouse alveolar macrophages. Expression levels of the above miRNAs alveolar macrophages were determined by qPCR and compared with those in bone marrow cells. Values are presented as mean ±SEM (n = 8).
